# Sinensetin Induces Oxidative Stress‐Mediated Apoptosis in Non‐Small Cell Lung Cancer Cells by Suppressing Nrf2/ARE‐Dependent Antioxidant Defense

**DOI:** 10.1002/jbt.71139

**Published:** 2026-10-25

**Authors:** Junyan Li, Ruoyun He, Jiake He, Jian Ye, Yu Xie, Binfan Yuan

**Affiliations:** ^1^ Center of Geriatric Medicine Affiliated Hospital of Guangdong Medical University Zhanjiang Guangdong China; ^2^ Department of Otolaryngology Affiliated Hospital of Guangdong Medical University Zhanjiang Guangdong China; ^3^ Department of Respiratory and Critical Care Medicine Affiliated Hospital of Guangdong Medical University Zhanjiang Guangdong China

**Keywords:** antioxidant defense, apoptosis, non‐small cell lung cancer, Nrf2/ARE, sinensetin

## Abstract

Non‐small cell lung cancer (NSCLC) cells frequently depend on Nrf2/ARE‐driven antioxidant programs to maintain redox balance and evade apoptosis. This study investigated whether sinensetin, a naturally occurring polymethoxylated flavone, reduces NSCLC cell survival by interfering with this cytoprotective pathway. A549 and H1299 NSCLC cells and normal BEAS‐2B bronchial epithelial cells were exposed to sinensetin. Cell viability was measured by MTT assay, apoptosis was quantified by Annexin V‐FITC/PI flow cytometry, and pathway‐ and apoptosis‐related proteins were analyzed by western blotting. Nrf2 distribution was evaluated by nuclear/cytoplasmic fractionation, ARE‐dependent transcription was assessed using a dual‐luciferase reporter system, and Nrf2 was silenced by shRNA. Intracellular ROS was measured by DCFH‐DA, antioxidant rescue was assessed with N‐acetyl‐l‐cysteine (NAC), Nrf2/ARE‐related proteins were examined in BEAS‐2B cells, and Nrf2 overexpression was used as a rescue approach in A549 cells. Sinensetin inhibited NSCLC cell growth, increased apoptosis, reduced total and nuclear Nrf2, decreased ARE reporter activity, and downregulated HO‐1 and NQO1. Sinensetin also increased intracellular ROS, whereas NAC partially reduced ROS accumulation, restored cell viability, and decreased apoptosis. BEAS‐2B cells showed comparatively modest Nrf2/ARE pathway changes. Nrf2 overexpression partially restored HO‐1 and NQO1, reduced apoptotic signaling, and attenuated sinensetin‐induced loss of viability and apoptosis. These results support a model in which sinensetin promotes oxidative stress‐mediated apoptosis in NSCLC cells, at least in part, through suppression of Nrf2/ARE‐dependent antioxidant defense.

## Graphical abstract

Sinensetin suppresses Nrf2/ARE signaling, increases intracellular ROS, and promotes mitochondrial apoptosis in A549 NSCLC cells. Partial rescue by NAC and Nrf2 overexpression supports a functional role for oxidative stress and Nrf2 suppression in the observed anticancer response.

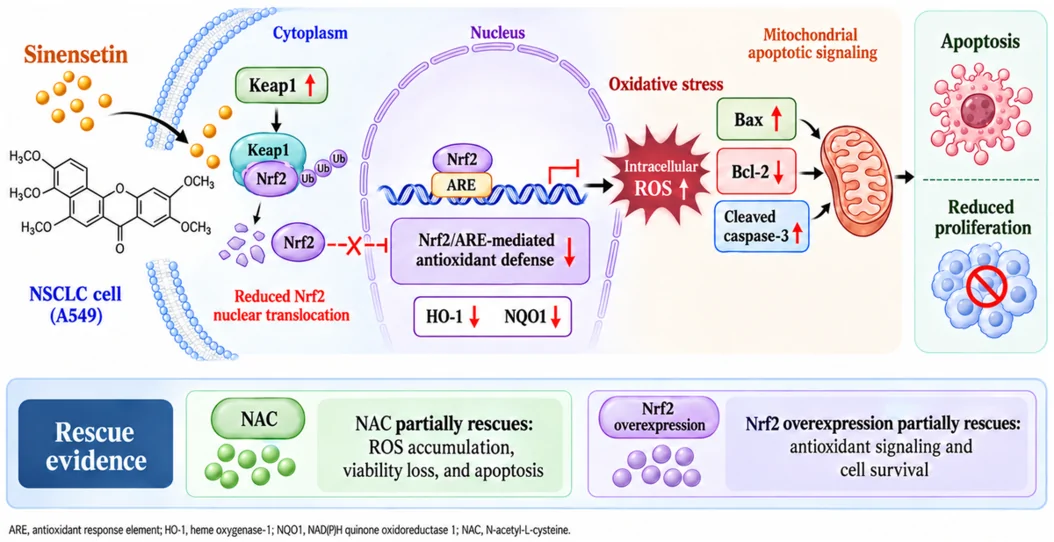

## 1 Introduction

Lung cancer remains a major challenge in global oncology because of its high incidence and mortality. Recent worldwide estimates reported approximately 2.5 million newly diagnosed lung cancer cases and more than 1.8 million deaths in 2022, confirming its position as one of the most frequently diagnosed and most fatal cancers [1, 2, 3]. Non‐small cell lung cancer (NSCLC) is the dominant pathological type, representing nearly 80%–85% of lung cancer cases, and includes adenocarcinoma, squamous cell carcinoma, and large cell carcinoma [3, 4]. Despite substantial progress in surgical treatment, chemotherapy, radiotherapy, molecular targeted therapy, and immune checkpoint‐based therapy, clinical outcomes remain unsatisfactory for many patients because diagnosis often occurs at an advanced stage and because recurrence, metastasis, and drug resistance frequently develop during treatment [5]. For this reason, natural compounds with antitumor activity and relatively low toxicity continue to attract attention as possible sources of new therapeutic candidates for NSCLC [6].

Nuclear factor erythroid 2‐related factor 2 (Nrf2) is an important transcriptional regulator involved in cellular redox balance, detoxification, and stress adaptation. Under normal conditions, Kelch‐like ECH‐associated protein 1 (Keap1) binds Nrf2 in the cytoplasm and promotes its ubiquitination and proteasomal degradation. In response to oxidative or electrophilic stimulation, Nrf2 dissociates from Keap1‐mediated control, accumulates in the nucleus, and activates antioxidant response element (ARE)‐dependent transcription. This process induces several cytoprotective genes, including heme oxygenase‐1 (HO‐1) and NAD(P)H quinone oxidoreductase 1 (NQO1) [7, 8]. Although this pathway is beneficial for protecting non‐malignant cells from oxidative injury and DNA damage, persistent Nrf2 activation can be advantageous for cancer cells. By enhancing antioxidant capacity and cellular defense, Nrf2 may support tumor growth, inhibit apoptosis, and reduce the response to anticancer treatment [9, 10, 11, 12]. In NSCLC, abnormal activation of the Nrf2/ARE pathway has been associated with apoptosis resistance and therapy resistance, indicating that blockade of this pathway may weaken tumor cell survival mechanisms [13, 14, 15, 16, 17, 18, 19]. Previous studies have shown that certain flavonoids, such as luteolin, can suppress Nrf2/ARE signaling in A549 cells and enhance sensitivity to anticancer drugs [16]. However, whether sinensetin regulates Nrf2/ARE‐dependent antioxidant defense and apoptosis in NSCLC cells remains unclear.

Sinensetin is a polymethoxylated flavone found in citrus peel and *Orthosiphon aristatus*, and it has been reported to possess anti‐inflammatory, antioxidant, anti‐angiogenic, and anticancer activities [20, 21]. Increasing evidence suggests that sinensetin affects multiple signaling pathways involved in tumor progression. For example, previous studies showed that sinensetin inhibited breast cancer cell progression by suppressing Wnt/β‐catenin signaling, reduced angiogenesis in liver cancer through regulation of the VEGF/VEGFR2/AKT pathway, and impaired NSCLC proliferation, migration, and tumor growth by inactivating AKT/β‐catenin signaling [22, 23, 24]. More recently, sinensetin was identified as a selective inhibitor of mitogen‐activated protein kinase kinase 6 (MKK6), and this effect was linked to reduced proliferation and clonogenic growth of NSCLC cells [25]. These findings indicate that sinensetin may exert anticancer effects through coordinated regulation of several tumor‐related pathways.

The influence of sinensetin on Nrf2 signaling appears to depend on the biological context. In carbon tetrachloride‐induced oxidative injury, sinensetin has been reported to activate Nrf2 and enhance antioxidant protection, suggesting a cytoprotective role in non‐tumor injury models [26]. However, in established cancer cells, sustained Nrf2/ARE activity may help tumor cells tolerate oxidative stress and resist anticancer treatment [9, 10, 11, 12]. Therefore, whether sinensetin modulates Nrf2/ARE‐mediated antioxidant defense in NSCLC cells and whether this regulation contributes to apoptosis induction remain to be clarified. In this study, we examined the effects of sinensetin on NSCLC cell proliferation and apoptosis and investigated their association with Nrf2/ARE‐mediated antioxidant defense.

## 2 Materials and Methods

### 2.1 Materials and Chemicals

Sinensetin with a purity greater than 98% was obtained from MedChemExpress (Monmouth Junction, NJ, USA). Its molecular weight is 372.37 g/mol; therefore, 50 and 100 μg/mL correspond to approximately 134.3 and 268.6 μM, respectively. MTT was supplied by Sigma‐Aldrich (St. Louis, MO, USA). Primary antibodies against β‐actin, GAPDH, Histone H3, Bax, Bcl‐2, cleaved caspase‐3, Keap1, Nrf2, NQO1, and HO‐1, together with HRP‐conjugated secondary antibodies, DCFH‐DA, and *N*‐acetyl‐l‐cysteine (NAC), were purchased from Santa Cruz Biotechnology (Santa Cruz, CA, USA). Plasmids encoding Nrf2 shRNA, the non‐targeting shRNA control, an Nrf2 overexpression construct, and the corresponding empty‐vector control were obtained from OriGene Technologies Inc. (Rockville, MD, USA). Lipofectamine 2000 was purchased from Invitrogen (Carlsbad, CA, USA). The Nuclear and Cytoplasmic Protein Extraction Kit was supplied by Sangon Biotech (Shanghai, China), and the Dual‐Luciferase Reporter Assay System was supplied by Promega (Madison, WI, USA). Other chemicals and reagents were analytical grade.

### 2.2 Cell Lines and Culture Conditions

A‐549 (A549; human, male, lung adenocarcinoma; RRID: CVCL_0023), NCI‐H1299 (H1299; human, male, lung large cell carcinoma derived from a metastatic lymph node; RRID: CVCL_0060), and BEAS‐2B (human, male, bronchial epithelial cell line derived from lung bronchial epithelium; RRID: CVCL_0168) were obtained from the Cell Bank of the Chinese Academy of Sciences (Shanghai, China) in 2024. According to current Cellosaurus records, these cell lines are not listed as misidentified cell lines. Cell line identity was confirmed by short tandem repeat (STR) profiling, and all cultures were confirmed to be free of mycoplasma contamination before use in the experiments. A549 and H1299 cells were maintained in DMEM containing 10% fetal bovine serum, 100 units/mL penicillin, and 0.1 mg/mL streptomycin. BEAS‐2B cells were maintained in DMEM/F12 medium according to the supplier's instructions. All cultures were incubated at 37°C in a humidified atmosphere containing 5% CO_2_.

### 2.3 Transfection of A549 Cells With Nrf2‐shRNA

A549 cells were selected for mechanistic experiments because they showed relatively high basal Nrf2 expression and greater sensitivity to sinensetin treatment. Nrf2 knockdown was performed using an shRNA‐based approach, as previously described with minor modifications [14]. Briefly, cells were seeded in 6‐well plates at a density of 6 × 10^5^ cells per well and allowed to grow to approximately 70%–90% confluence. They were then transfected with either the Nrf2‐shRNA plasmid or the non‐targeting control shRNA plasmid using Lipofectamine 2000 following the manufacturer's instructions. After 48 h, the transfection medium was replaced with fresh complete medium, and the cells were cultured for another 48 h. The efficiency of Nrf2 silencing was verified by RT‐qPCR and western blotting before the cells were used for further experiments.

### 2.4 MTT Assay

MTT assay was performed as previously described with minor modifications [25]. Briefly, A549, H1299, and BEAS‐2B cells were seeded in 96‐well plates and allowed to attach overnight. Cells were then exposed to sinensetin at 0–100 μg/mL (0–268.6 μM) for 24, 48, or 72 h; the tested concentrations of 5, 10, 25, 50, and 100 μg/mL correspond to approximately 13.4, 26.9, 67.1, 134.3, and 268.6 μM, respectively. At the end of treatment, the medium was replaced with fresh medium containing 0.5 mg/mL MTT, and plates were incubated for 4 h at 37°C. After removal of the MTT‐containing medium, 150 μL DMSO was added to each well to dissolve formazan crystals. Absorbance was read at 490 nm using a microplate reader after gentle shaking for 10 min. Viability was calculated as a percentage of the untreated control. Experiments were repeated 3 times.

### 2.5 Apoptosis Assay

Apoptosis was assessed by Annexin V‐FITC/PI staining and flow cytometry, as previously described with minor modifications [27]. Briefly, A549 and H1299 cells were treated with 50 μg/mL (134.3 μM) or 100 μg/mL (268.6 μM) sinensetin for 48 h, collected, washed twice with cold PBS, and resuspended in 1× binding buffer. Each sample was incubated with 5 μL Annexin V‐FITC and 10 μL PI for 15 min at room temperature in the dark and then analyzed immediately by flow cytometry. Cells negative for both Annexin V and PI were considered viable. Annexin V‐positive/PI‐negative cells were defined as early apoptotic cells, Annexin V‐positive/PI‐positive cells as late apoptotic cells, and Annexin V‐negative/PI‐positive cells as necrotic cells. Total apoptosis was calculated from the Annexin V‐positive population.

### 2.6 Luciferase Reporter Assay

ARE‐luciferase reporter assay was performed to evaluate Nrf2/ARE transcriptional activity, as previously described with minor modifications [16]. Briefly, A549 cells were seeded into 6‐well plates and transfected at approximately 50%–70% confluence with an ARE‐firefly luciferase reporter plasmid together with a Renilla luciferase internal‐control plasmid using Lipofectamine 2000. After overnight recovery, cells were treated with 50 μg/mL (134.3 μM) or 100 μg/mL (268.6 μM) sinensetin for 24 h. Firefly and Renilla luciferase activities were measured with the Dual‐Luciferase Reporter Assay System. ARE reporter activity was expressed as the ratio of firefly to Renilla luciferase activity to normalize for differences in transfection efficiency, and each experiment was performed in triplicate.

### 2.7 Western Blotting Analysis

Western blotting was performed as previously described with minor modifications [16]. Total, cytoplasmic, and nuclear protein fractions were prepared for western blot analysis. Cytoplasmic and nuclear proteins were isolated using the Nuclear and Cytoplasmic Protein Extraction Kit, whereas total proteins were extracted with RIPA buffer supplemented with protease and phosphatase inhibitors. Lysates were centrifuged, and supernatants were stored at −80°C until analysis. Protein concentrations were measured with the BCA method. Equal quantities of protein, generally 30 μg per lane, were resolved by 10%–12% SDS‐PAGE and transferred to PVDF membranes. After blocking for 1 h at room temperature with 5% non‐fat milk in PBS or TBST containing 0.1% Tween‐20, membranes were incubated overnight at 4°C with antibodies against Keap1, Nrf2, HO‐1, NQO1, Bax, Bcl‐2, cleaved caspase‐3, β‐actin, GAPDH, or Histone H3. Membranes were then incubated with HRP‐linked secondary antibodies for 1 h at room temperature. GAPDH or β‐actin served as the loading control for total and cytoplasmic proteins, and Histone H3 served as the nuclear loading control. Bands were detected using enhanced chemiluminescence.

### 2.8 Total RNA Extraction and RT‐qPCR

Total RNA extraction and RT‐qPCR analysis were performed as previously described with minor modifications [28]. Total RNA was isolated from A549 cells using TRIzol reagent after the indicated treatments. RNA concentration and purity were assessed by spectrophotometry, and cDNA was generated with a reverse transcription kit. Quantitative PCR was carried out with SYBR‐Green PCR Mix on a real‐time PCR system. Primer sequences were as follows: Nrf2 forward, 5′‐ACACGGTCCACAGCTCATC‐3′ and reverse, 5′‐TGTCAATCAAATCCATGTCCTG‐3′; NQO1 forward, 5′‐GGTTTGAGCGAGTGTTCATAGG‐3′ and reverse, 5′‐GCAGAGAGTACATGGAGCCAC‐3′; HO‐1 forward, 5′‐AACTTTCAGAAGGGCCAGGT‐3′ and reverse, 5′‐CTGGGCTCTCCTTGTTGC‐3′; GAPDH forward, 5′‐CGGAGTCAACGGATTTGGTCGTAT‐3′ and reverse, 5′‐AGCCTTCTCCATGGTGGTGAAGAC‐3′. The amplification program consisted of 95°C for 10 min, followed by 45 cycles of 95°C for 5 s, 60°C for 30 s, and 72°C for 30 s. Relative expression was calculated by the 2^‐ΔΔCq method with GAPDH as the internal control. All assays were conducted in triplicate.

### 2.9 Intracellular ROS Detection and NAC Rescue

Intracellular ROS was assessed in A549 cells using DCFH‐DA staining. Cells were assigned to Control, Sinensetin, NAC, or Sinensetin + NAC groups. Cells in the Sinensetin and Sinensetin + NAC groups were treated with 100 μg/mL (268.6 μM) sinensetin for 48 h. For rescue experiments, cells were pretreated with 5 mM NAC for 1 h before sinensetin exposure. After 48 h of sinensetin treatment, intracellular ROS‐associated fluorescence was measured and expressed relative to the untreated control. In parallel, cell viability was evaluated by MTT assay, and apoptosis was measured by Annexin V‐FITC/PI flow cytometry to determine whether NAC attenuated the cellular effects of sinensetin.

### 2.10 Nrf2/ARE Pathway Analysis in BEAS‐2B Cells

To assess whether sinensetin produced comparable pathway changes in non‐malignant bronchial epithelial cells, BEAS‐2B cells were treated with 0, 50 μg/mL (134.3 μM), or 100 μg/mL (268.6 μM) sinensetin for 48 h. Total protein lysates were then prepared and analyzed by western blotting for Nrf2, HO‐1, and NQO1, with β‐actin used as the loading control.

### 2.11 Nrf2 Overexpression Rescue in A549 Cells

A549 cells were transiently transfected with an Nrf2 overexpression plasmid or the corresponding empty‐vector control using Lipofectamine 2000. After recovery, cells were assigned to Vector, Vector + Sinensetin, Nrf2‐OE, and Nrf2‐OE + Sinensetin groups. Cells in the sinensetin‐treated groups were exposed to 100 μg/mL (268.6 μM) sinensetin for 48 h. Nrf2, HO‐1, NQO1, Bax, Bcl‐2, and cleaved caspase‐3 were analyzed by western blotting. Cell viability and apoptosis were assessed by MTT assay and Annexin V‐FITC/PI flow cytometry, respectively. Rescue was defined as a partial reversal of the molecular or functional changes induced by sinensetin in vector‐transfected cells.

### 2.12 Statistical Analysis

Data are shown as mean ± standard deviation (SD) from at least three independent experiments. Statistical analysis was performed with GraphPad Prism 8. Student's *t*‐test was used for two‐group comparisons, and one‐way ANOVA followed by Tukey's multiple‐comparisons test was used for comparisons among more than two groups. Statistical significance was defined as *p* < 0.05.

## 3 Results

### 3.1 Sinensetin Suppresses Proliferation of NSCLC Cells

To assess the growth‐inhibitory activity of sinensetin, A549, H1299, and BEAS‐2B cells were exposed to 0–100 μg/mL (0–268.6 μM) sinensetin for 24, 48, or 72 h. The concentration series of 5, 10, 25, 50, and 100 μg/mL corresponds to approximately 13.4, 26.9, 67.1, 134.3, and 268.6 μM, respectively. MTT analysis showed a concentration‐dependent reduction in viability in the NSCLC cell lines, with the most pronounced response observed in A549 cells after 48 h at 50 μg/mL (134.3 μM) and 100 μg/mL (268.6 μM) (Figure 1A, *p *< 0.05). In A549 cells, viability declined from 100 ± 8.9% in the untreated control group to 65.1 ± 5.9% and 48.4 ± 4.3% after treatment with 50 and 100 μg/mL sinensetin, respectively. H1299 cells showed a less marked response, with significant inhibition mainly at 50 and 100 μg/mL after 48 and 72 h (Figure 1B, *p *< 0.05). In contrast, BEAS‐2B cell viability was not obviously altered by sinensetin under the same conditions (Figure 1C). These results indicate that, under the tested conditions, sinensetin exerted a greater inhibitory effect on NSCLC cells than on BEAS‐2B cells, with A549 cells showing the strongest response. The lower‐concentration viability data should not be interpreted as evidence that Nrf2/ARE signaling is already suppressed at those concentrations, because pathway measurements were performed at 50 and 100 μg/mL.

**Figure 1:**
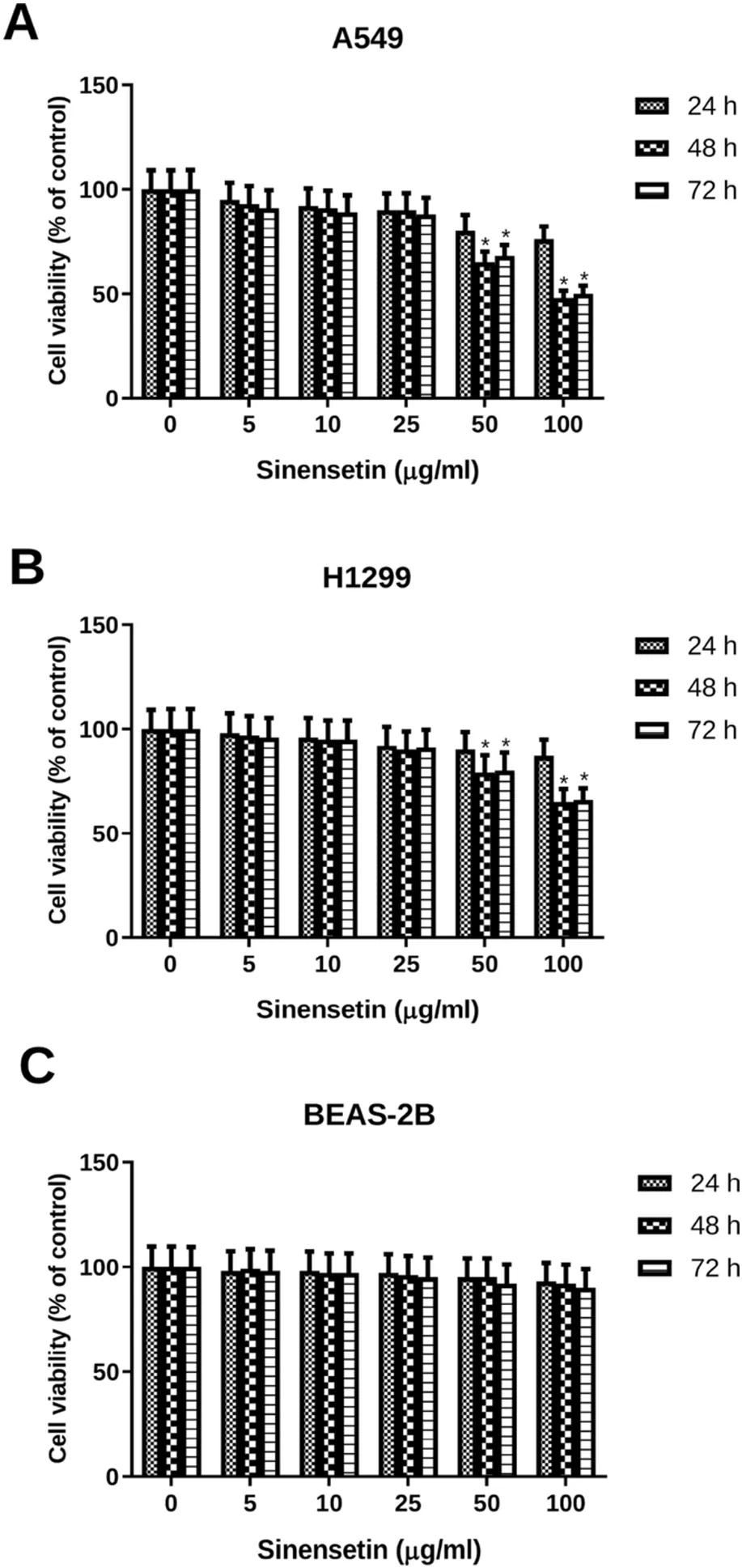
Effects of sinensetin on NSCLC and normal bronchial epithelial cell viability. A549, H1299, and BEAS‐2B cells were exposed to the indicated concentrations of sinensetin for 24, 48, or 72 h, and viability was measured by MTT assay. Concentrations of 5, 10, 25, 50, and 100 μg/mL correspond to approximately 13.4, 26.9, 67.1, 134.3, and 268.6 μM, respectively. Results are shown as mean ± SD from three independent experiments. *p < 0.05 versus control.

### 3.2 Sinensetin Induces Apoptosis of NSCLC Cells

Because sinensetin reduced NSCLC cell viability, Annexin V‐FITC/PI staining was used to determine whether apoptosis contributed to this effect. A549 and H1299 cells were treated with 50 μg/mL (134.3 μM) or 100 μg/mL (268.6 μM) sinensetin for 48 h before flow cytometric analysis. In A549 cells, both concentrations clearly increased the apoptotic population compared with untreated controls (Figure 2A, *p *< 0.05). Consistent with the viability results, H1299 cells also displayed a significant increase in apoptosis after exposure to 100 μg/mL (268.6 μM) sinensetin for 48 h (Figure 2B, *p* < 0.05). Thus, apoptosis induction was involved in the antiproliferative effect of sinensetin in NSCLC cells.

**Figure 2:**
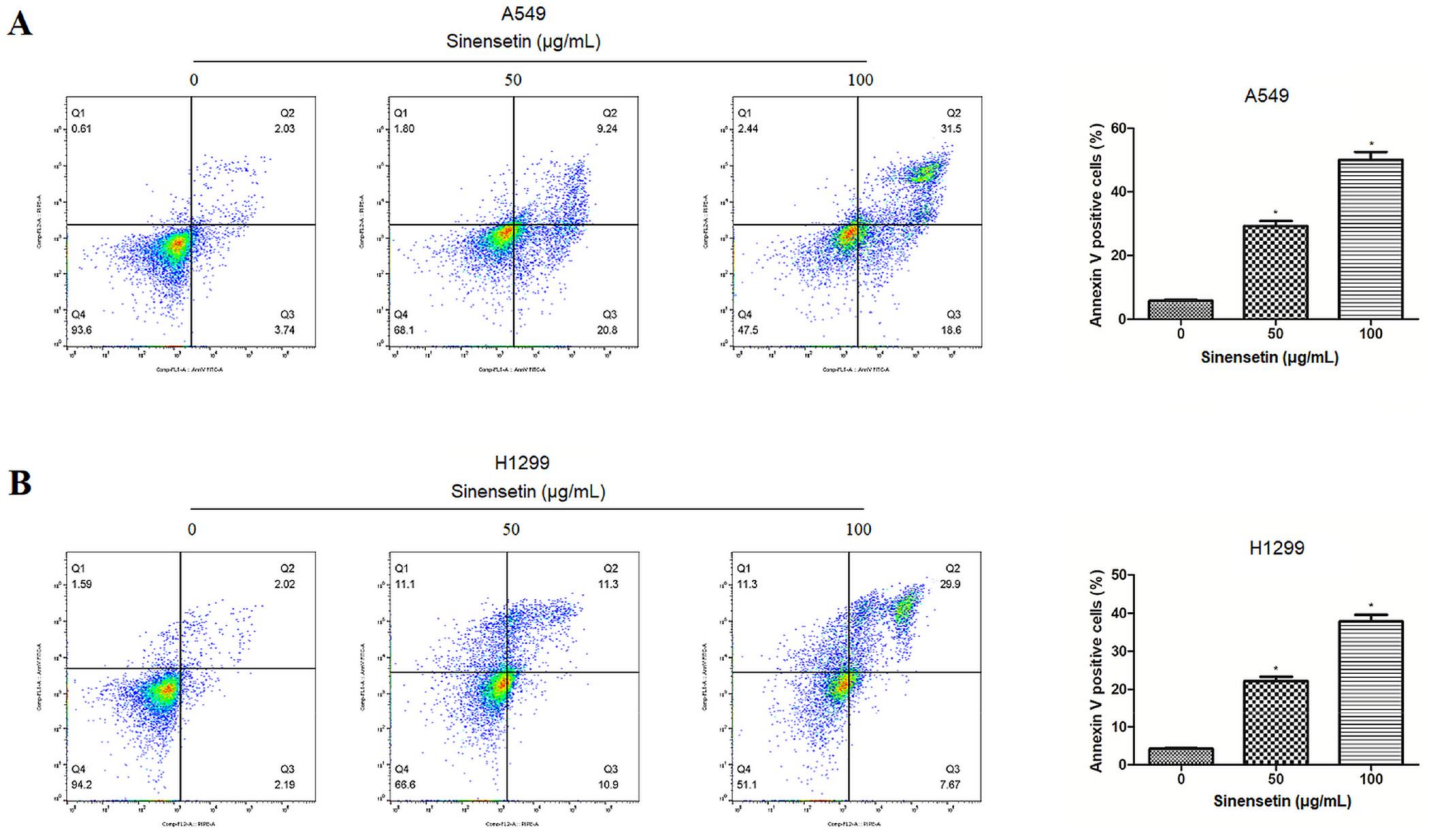
Apoptosis induction by sinensetin in NSCLC cells. A549 and H1299 cells were treated with 50 μg/mL (134.3 μM) or 100 μg/mL (268.6 μM) sinensetin for 48 h. Apoptotic cells were quantified by Annexin V‐FITC/PI staining followed by flow cytometry. Values represent mean ± SD from three independent experiments. *p < 0.05 versus control.

### 3.3 Sinensetin Suppresses Nrf2/ARE‐Mediated Antioxidant Defense and Activates Apoptotic Signaling in A549 Cells

Given the relatively high basal Nrf2 expression in A549 cells, we further examined whether the Nrf2/ARE pathway was altered by sinensetin. Total, cytoplasmic, and nuclear Nrf2 protein levels, together with NQO1, HO‐1, Bax, Bcl‐2, cleaved caspase‐3, and Keap1 expression, were analyzed in A549 cells. As shown in Figure 3A,B, sinensetin at 50 μg/mL (134.3 μM) and 100 μg/mL (268.6 μM) reduced total Nrf2 expression relative to untreated cells (*p* < 0.05), while Keap1 expression increased (*p* < 0.05). Sinensetin was associated with cytoplasmic retention of Nrf2 and a concomitant reduction of nuclear Nrf2. ARE reporter analysis, normalized to the co‐transfected Renilla luciferase control, further showed that Nrf2/ARE transcriptional activity was significantly decreased after sinensetin treatment at both concentrations for 24 h (Figure 3C, *p* < 0.05 or *p* < 0.01). In parallel, Bax and cleaved caspase‐3 levels increased, whereas Bcl‐2 decreased. These findings show that sinensetin‐induced apoptosis in A549 cells was accompanied by suppression of Nrf2/ARE‐mediated antioxidant signaling.

**Figure 3:**
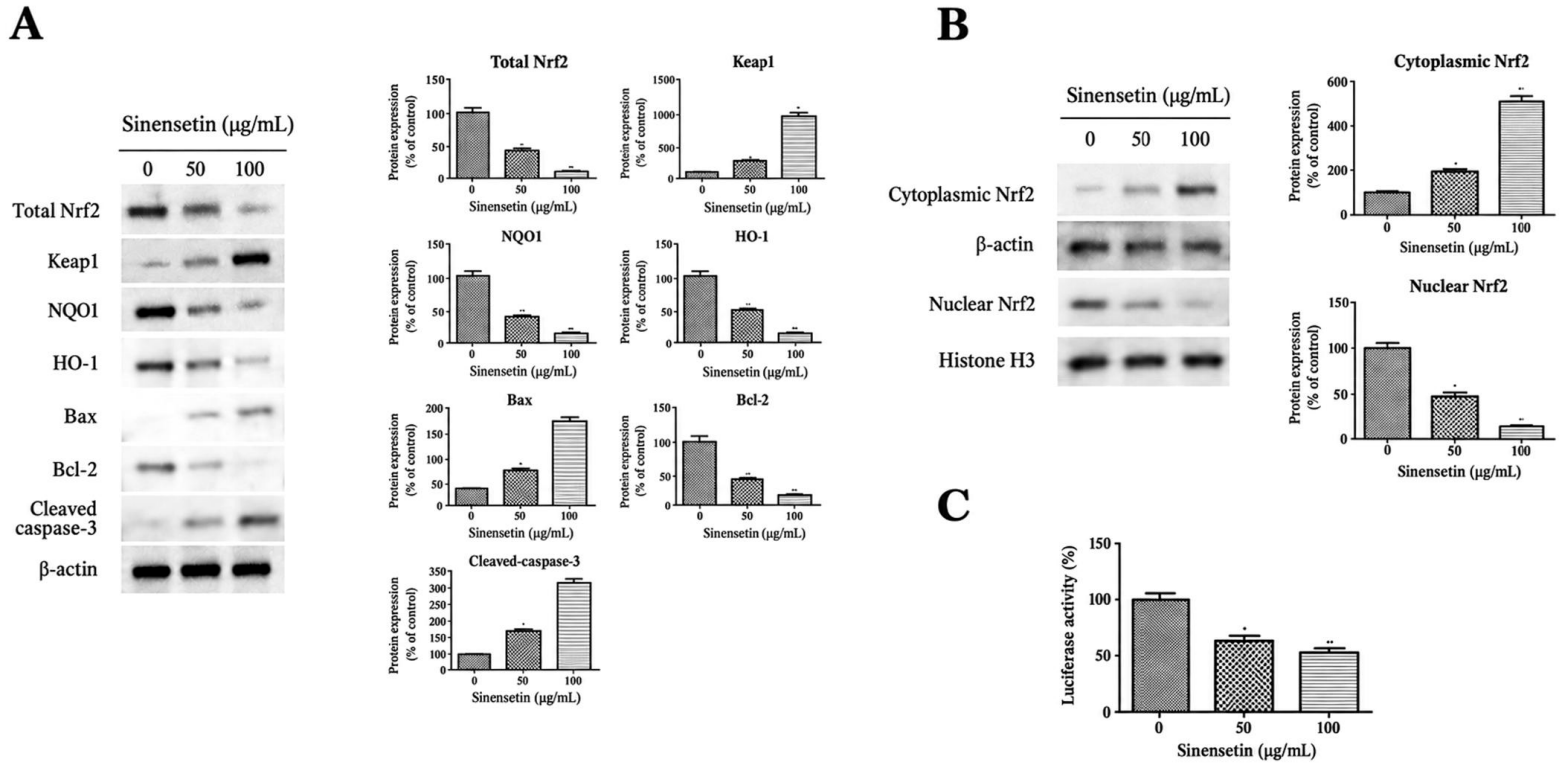
Regulation of Nrf2/ARE signaling and apoptotic proteins by sinensetin in A549 cells. (A, B) Protein levels of Keap1, total Nrf2, cytoplasmic Nrf2, nuclear Nrf2, HO‐1, NQO1, Bax, Bcl‐2, and cleaved caspase‐3 after treatment with 50 μg/mL (134.3 μM) or 100 μg/mL (268.6 μM) sinensetin for 48 h. (C) ARE‐luciferase reporter activity in A549 cells after treatment with 50 μg/mL (134.3 μM) or 100 μg/mL (268.6 μM) sinensetin for 24 h, normalized to Renilla luciferase activity. Data are expressed as mean ± SD from three independent experiments. *p < 0.05 and **p < 0.01 versus control.

### 3.4 Nrf2 Expression in NSCLC Cells and Validation of Nrf2 Knockdown

To clarify the relevance of Nrf2 in the response to sinensetin, Nrf2 expression was first compared between A549 and H1299 cells. As presented in Figure 4A–D, total and nuclear Nrf2 protein levels were lower in H1299 cells than in A549 cells. Considering both the stronger response of A549 cells to sinensetin and their higher Nrf2 expression, subsequent mechanistic experiments focused mainly on A549 cells. A549 cells were then transfected with Nrf2‐shRNA. Compared with untreated and NC‐shRNA control cells, Nrf2‐shRNA‐transfected cells showed a marked reduction in Nrf2 mRNA and total Nrf2 protein expression (Figure 5), confirming efficient Nrf2 knockdown.

**Figure 4:**
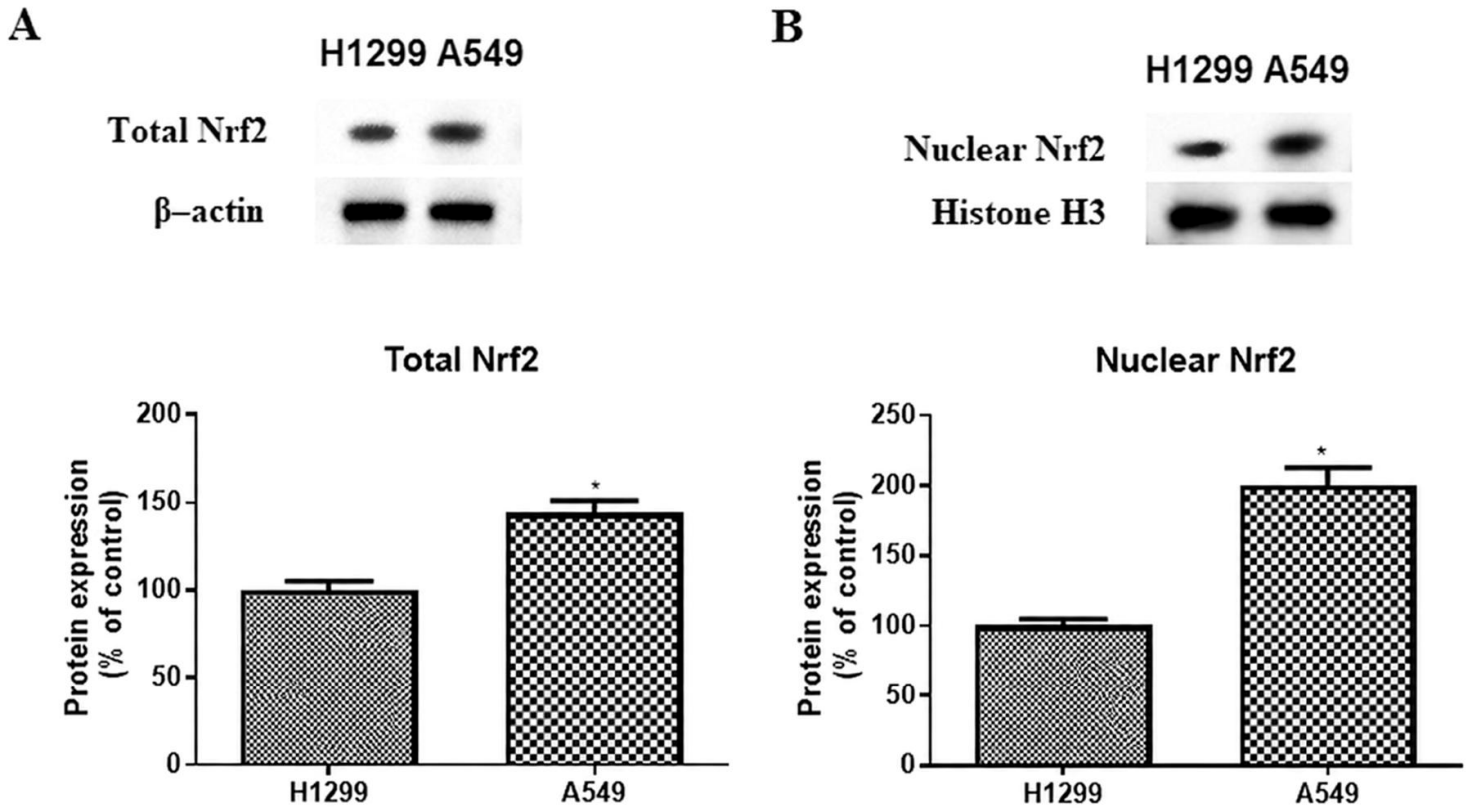
Comparison of basal Nrf2 expression between NSCLC cell lines. Total Nrf2 protein, nuclear Nrf2 protein, and Nrf2 mRNA levels were analyzed in A549 and H1299 cells. Results are presented as mean ± SD from three independent experiments. *p < 0.05 versus the indicated group.

**Figure 5:**
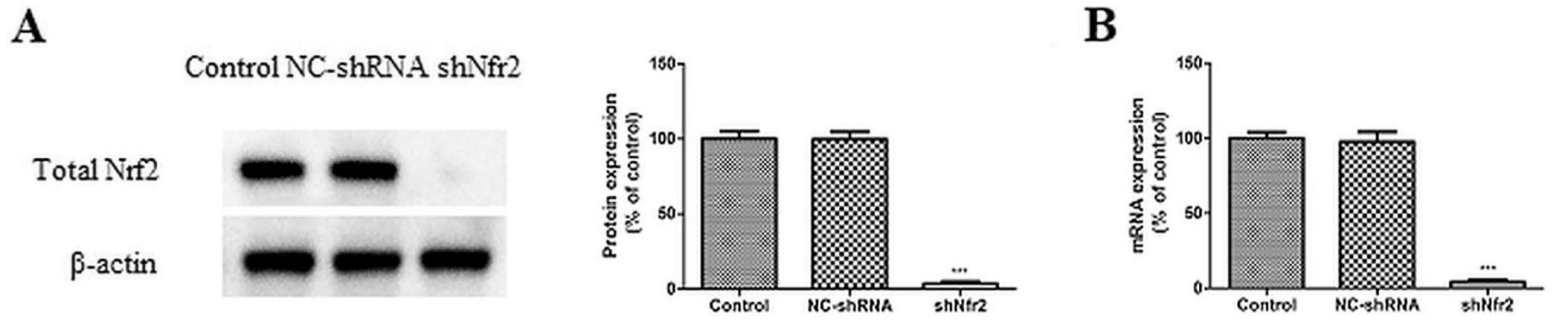
Verification of Nrf2 knockdown in A549 cells. A549 cells were transfected with Nrf2‐shRNA or non‐targeting control shRNA, and Nrf2 silencing was confirmed by western blotting and RT‐qPCR. Data are shown as mean ± SD from three independent experiments. ***p < 0.001 versus control.

### 3.5 Nrf2 Knockdown Modulates Antioxidant and Apoptotic Signaling in A549 Cells

We next assessed whether silencing Nrf2 altered antioxidant and apoptosis‐related proteins in A549 cells. As shown in Figure 6, Nrf2 knockdown reduced total Nrf2, HO‐1, NQO1, and Bcl‐2 expression, while increasing Keap1, Bax, and cleaved caspase‐3 levels. The increase in Keap1 was reproducibly observed in the three independent experiments. Overall, loss of Nrf2 weakened antioxidant defense and shifted the apoptotic balance toward cell death.

**Figure 6:**
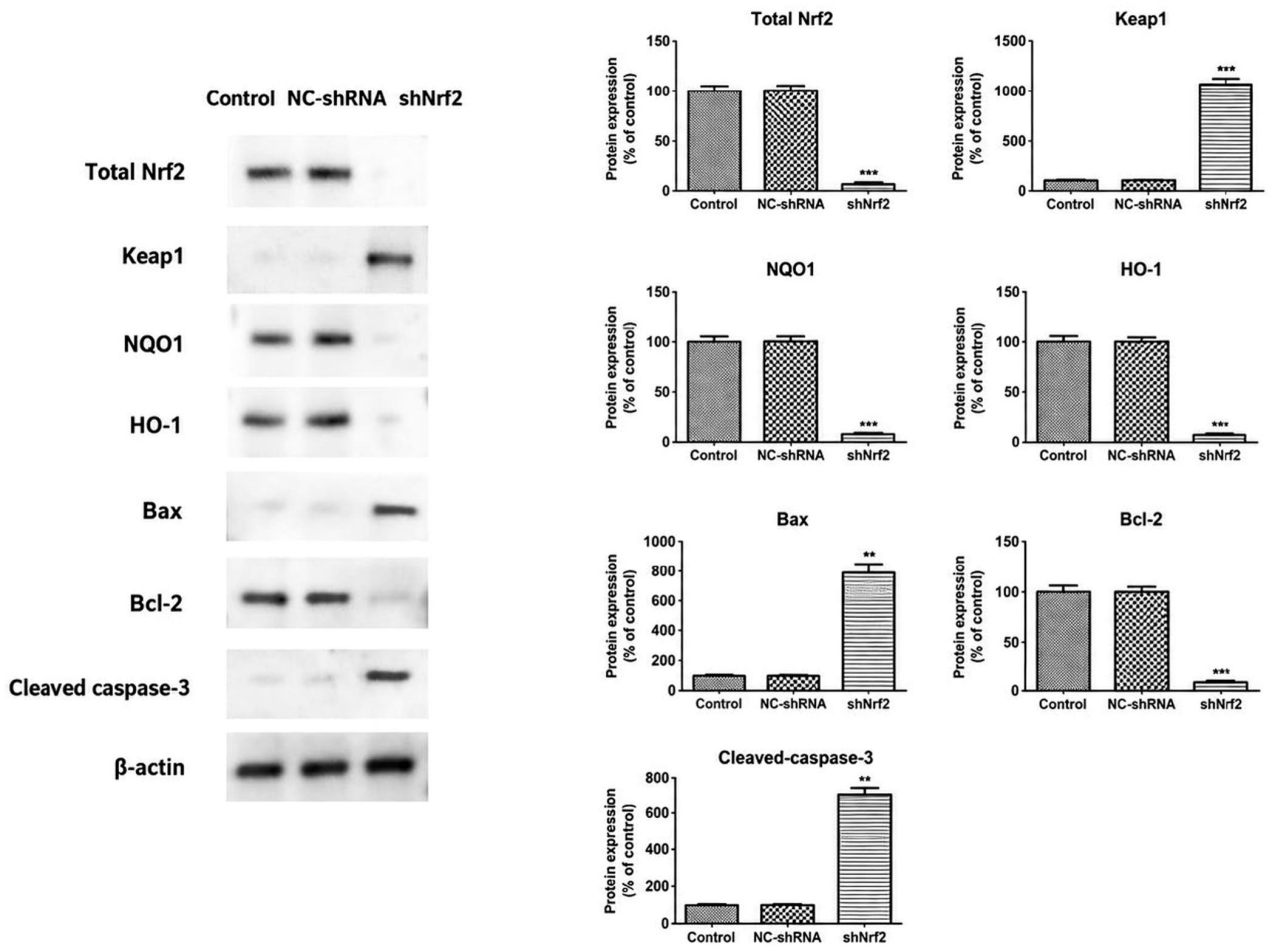
Effects of Nrf2 knockdown on antioxidant and apoptosis‐related signaling in A549 cells. Keap1, Nrf2, HO‐1, NQO1, Bax, Bcl‐2, and cleaved caspase‐3 protein levels were examined in control, NC‐shRNA, and Nrf2‐shRNA A549 cells. Results are presented as mean ± SD from three independent experiments. **p < 0.01 and ***p < 0.001 versus control.

### 3.6 Nrf2 Knockdown Suppresses Proliferation and Enhances Apoptosis of A549 Cells

Finally, we evaluated whether Nrf2 knockdown affected A549 cell viability and apoptosis. A549 cells expressing Nrf2‐shRNA showed lower viability and a higher proportion of apoptotic cells compared with normal A549 cells and control groups (*p* < 0.05, Figure 7). These results support the view that inhibition of Nrf2/ARE‐dependent antioxidant defense contributes, at least in part, to the antitumor activity of sinensetin in A549 cells.

**Figure 7:**
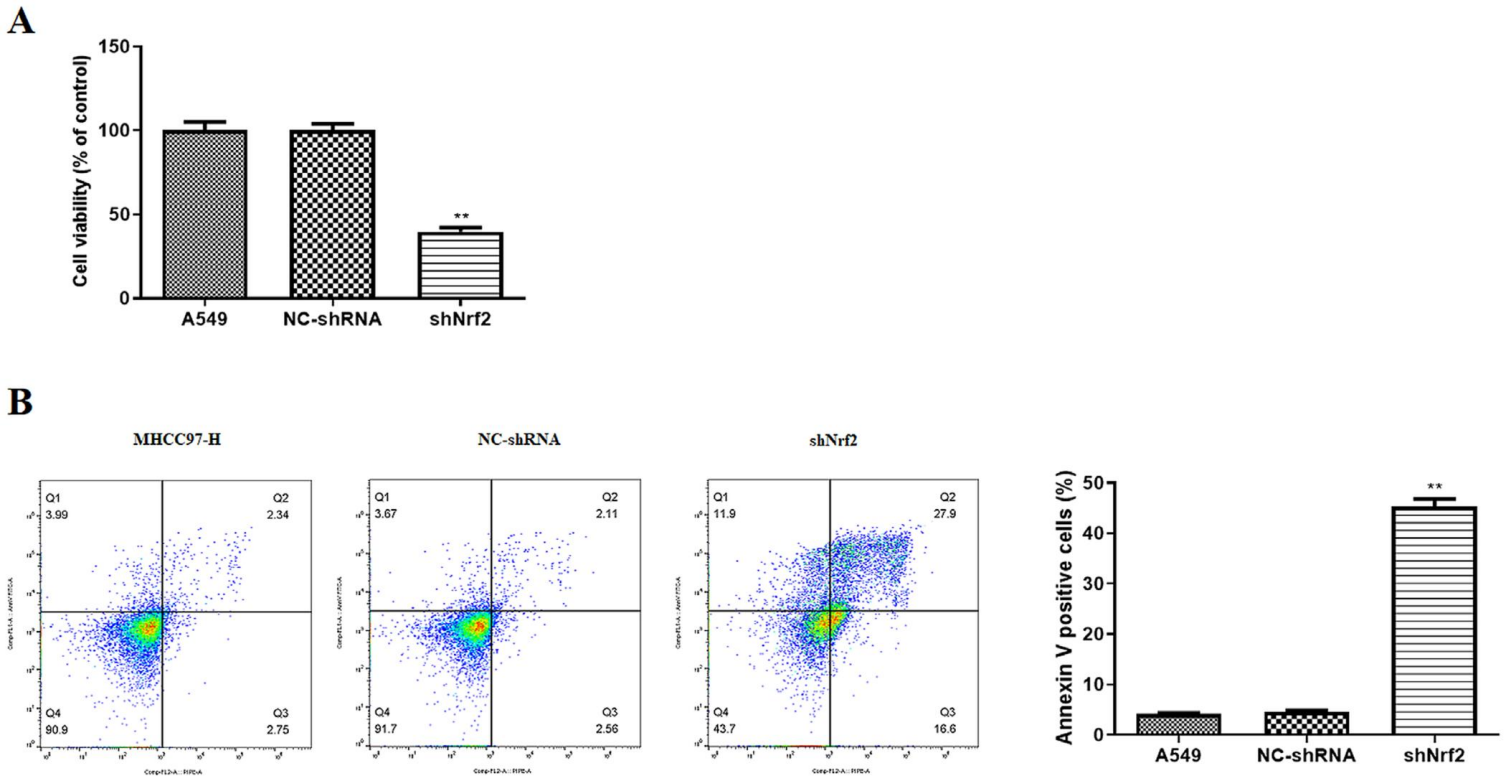
Functional effects of Nrf2 knockdown on A549 cell survival and apoptosis. (A) Cell viability was determined by MTT assay. (B) Apoptosis was analyzed by Annexin V‐FITC/PI staining and flow cytometry. Data are expressed as mean ± SD from three independent experiments. *p < 0.05 versus control.

### 3.7 NAC Partially Attenuates Sinensetin‐Induced ROS Accumulation and Cell Injury in A549 Cells

To test whether oxidative stress contributes functionally to the response to sinensetin, intracellular ROS was measured and NAC was used as an antioxidant rescue treatment. As shown in Figure 8A,B, sinensetin markedly increased DCFH‐DA fluorescence and intracellular ROS compared with the control group. NAC alone had little effect on basal ROS, whereas NAC pretreatment significantly reduced the sinensetin‐induced increase in ROS. Consistent with this effect, NAC pretreatment significantly improved cell viability and reduced the proportion of Annexin V‐positive cells in sinensetin‐treated A549 cells (Figure 8C,D). These findings support a functional contribution of ROS accumulation to sinensetin‐induced apoptosis.

**Figure 8:**
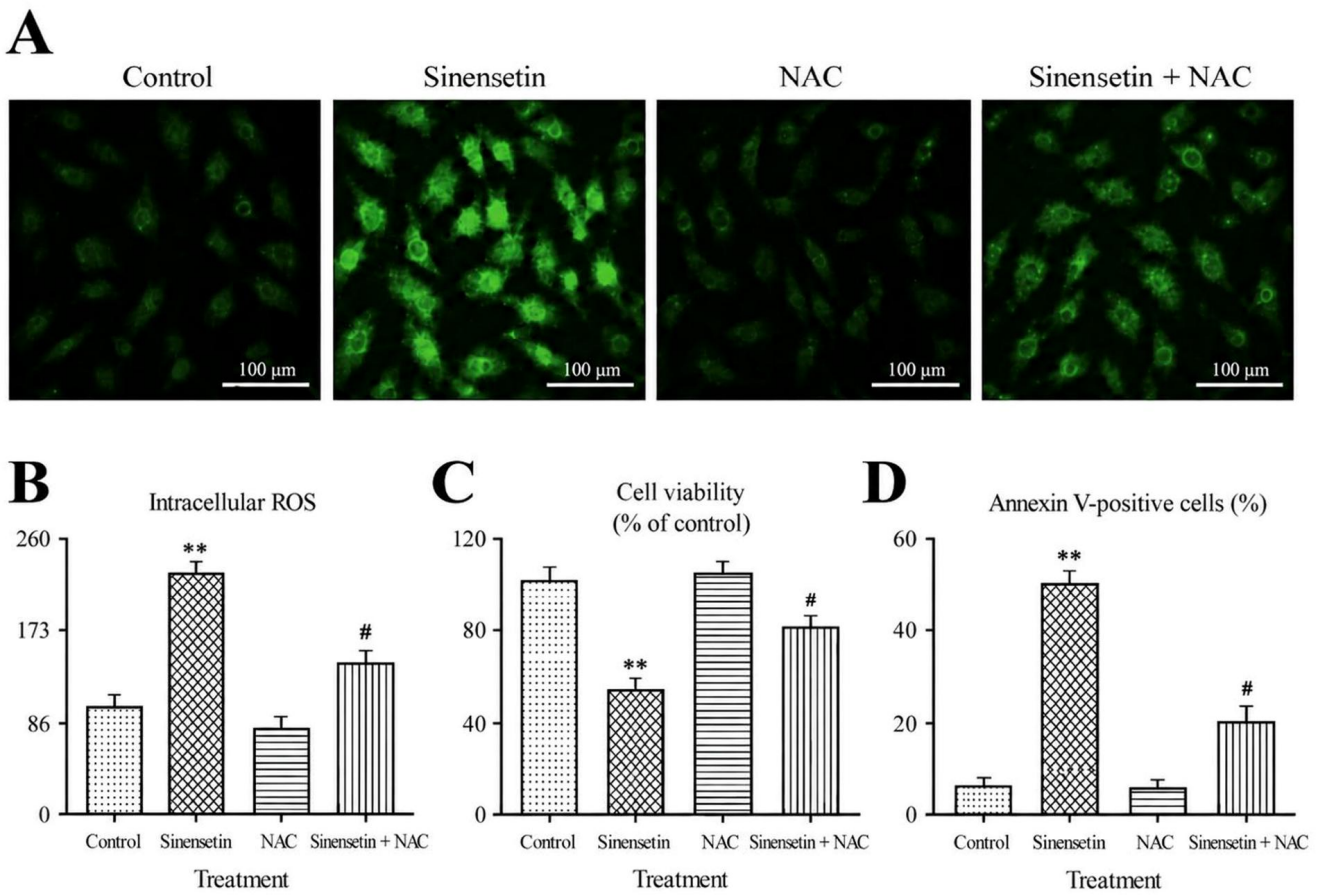
NAC partially attenuates sinensetin‐induced oxidative stress and apoptosis in A549 cells. A549 cells were pretreated with 5 mM NAC for 1 h and then exposed to 100 μg/mL (268.6 μM) sinensetin for 48 h. (A) Representative DCFH‐DA fluorescence images of Control, Sinensetin, NAC, and Sinensetin + NAC groups. (B) Quantification of intracellular ROS. (C) Cell viability determined by MTT assay. (D) Annexin V‐positive cells measured by flow cytometry. Data are expressed as mean ± SD from three independent experiments. **p < 0.01 versus Control; #*p* < 0.05 versus Sinensetin.

### 3.8 Sinensetin Produces More Limited Nrf2/ARE Pathway Changes in BEAS‐2B Cells

The effect of sinensetin on Nrf2/ARE‐related proteins was next examined in BEAS‐2B cells. As shown in Figure 9, treatment with 50 μg/mL (134.3 μM) or 100 μg/mL (268.6 μM) sinensetin for 48 h produced modest decreases in Nrf2, HO‐1, and NQO1 expression; however, these changes did not reach statistical significance. In contrast, more pronounced suppression of these proteins was observed in A549 cells under the tested conditions. Together with the limited effect of sinensetin on BEAS‐2B cell viability shown in Figure 1C, these findings indicate that the Nrf2/ARE pathway response to sinensetin was less pronounced in non‐malignant bronchial epithelial cells.

**Figure 9:**
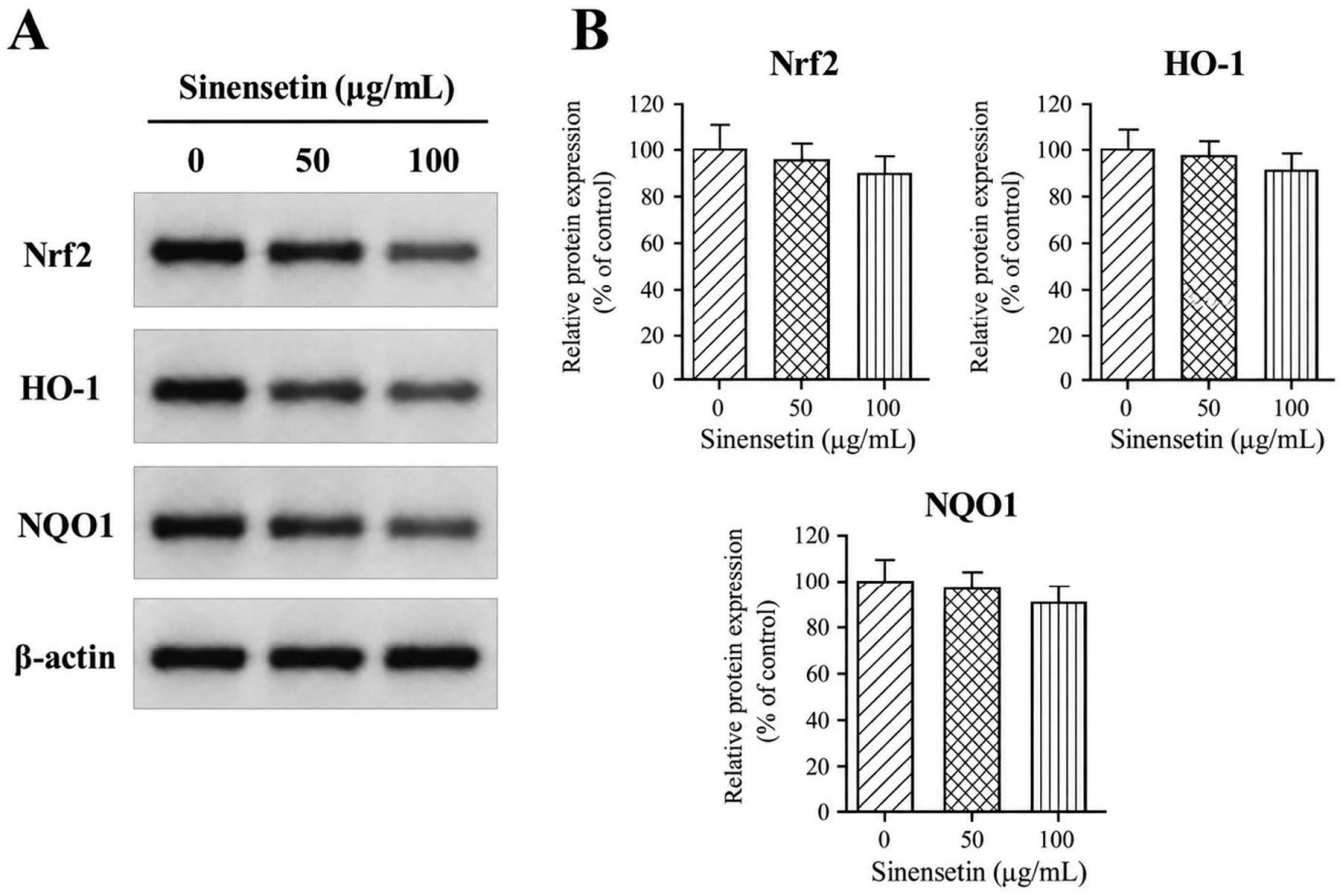
Effects of sinensetin on Nrf2/ARE‐related proteins in BEAS‐2B cells. (A) Representative western blot bands for Nrf2, HO‐1, NQO1, and β‐actin after treatment with 0, 50 μg/mL (134.3 μM), or 100 μg/mL (268.6 μM) sinensetin for 48 h. (B) Densitometric quantification of protein expression. Data are expressed as mean ± SD from three independent experiments.

### 3.9 Nrf2 Overexpression Partially Rescues Sinensetin‐Induced Apoptotic Signaling in A549 Cells

To further examine whether Nrf2 suppression contributes causally to the cellular response, Nrf2 overexpression rescue experiments were performed. As shown in Figure 10A,B, Nrf2 overexpression increased Nrf2 abundance and was accompanied by higher HO‐1 and NQO1 expression relative to vector‐transfected cells treated with sinensetin. Nrf2 overexpression also attenuated Bax and cleaved caspase‐3 induction and partially restored Bcl‐2 expression. Functionally, Nrf2 overexpression partially restored cell viability and reduced apoptosis after sinensetin treatment (Figure 10C,D). The incomplete rescue indicates that Nrf2 suppression is an important component of the response but is unlikely to represent the only pathway involved.

**Figure 10:**
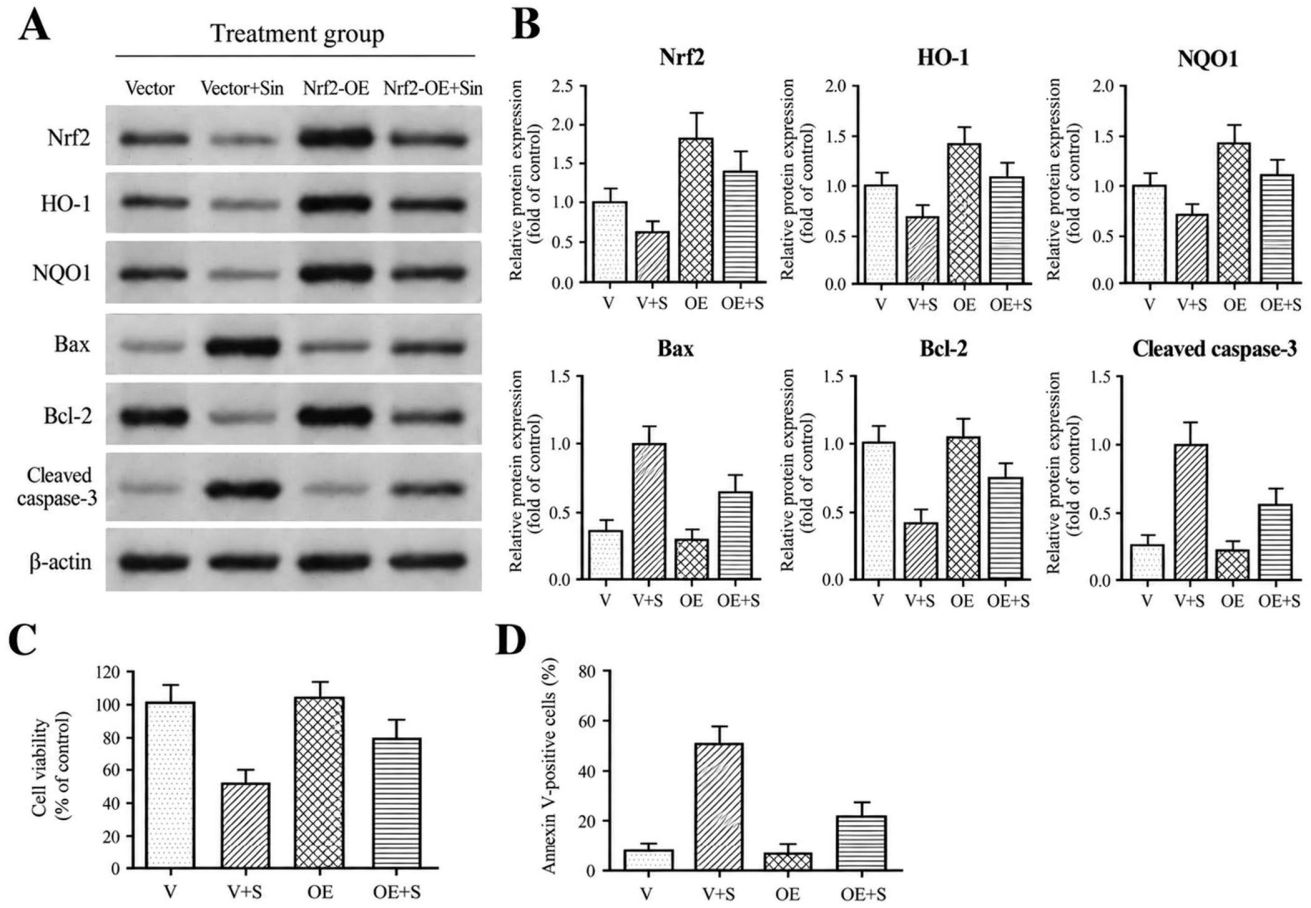
Nrf2 overexpression partially rescues sinensetin‐induced molecular and functional changes in A549 cells. A549 cells were treated with 100 μg/mL (268.6 μM) sinensetin for 48 h in the indicated vector and Nrf2‐overexpression groups. (A) Representative western blot bands for Nrf2, HO‐1, NQO1, Bax, Bcl‐2, cleaved caspase‐3, and β‐actin in Vector, Vector + Sinensetin, Nrf2‐OE, and Nrf2‐OE + Sinensetin groups. (B) Densitometric quantification of protein expression. (C) Cell viability. (D) Annexin V‐positive cells. Data are expressed as mean ± SD from three independent experiments.

## 4 Discussion

This study demonstrates that sinensetin suppresses NSCLC cell growth and promotes apoptotic cell death. Among the two NSCLC cell lines examined, A549 cells responded more strongly to sinensetin and were therefore used for most mechanistic experiments. Sinensetin reduced total and nuclear Nrf2, lowered ARE‐dependent reporter activity, and downregulated HO‐1 and NQO1 while increasing Bax and cleaved caspase‐3 and reducing Bcl‐2. Nrf2 knockdown reproduced many of these changes. Importantly, two complementary rescue approaches provide functional support for this interpretation: NAC partially reduced sinensetin‐induced ROS accumulation and apoptosis, while Nrf2 overexpression partially restored antioxidant signaling and cell survival. Together, these findings strengthen the interpretation that suppression of Nrf2/ARE‐dependent antioxidant defense contributes to oxidative stress‐mediated apoptosis in A549 cells.

Sinensetin, a polymethoxylated flavone present in citrus peel and *Orthosiphon aristatus*, has been associated with a broad range of biological activities, including anti‐inflammatory, antioxidant, anti‐angiogenic, and anticancer actions [20, 21]. Existing cancer studies suggest that sinensetin can act through multiple signaling pathways rather than through a single molecular mechanism. Reported targets include Wnt/β‐catenin signaling in breast cancer, VEGF/VEGFR2/AKT signaling in liver cancer angiogenesis, AKT/β‐catenin signaling in NSCLC, and MKK6‐dependent signaling in NSCLC cell proliferation and clonogenic survival [22, 23, 24, 25]. The present findings add Nrf2/ARE‐dependent antioxidant defense to the signaling processes associated with the anticancer activity of sinensetin in NSCLC cells.

Nrf2 is widely recognized as a master regulator of cellular antioxidant and detoxification responses. Under basal conditions, Keap1 promotes the cytoplasmic retention and degradation of Nrf2. Under stress conditions, Nrf2 accumulates in the nucleus and binds ARE sequences to stimulate transcription of genes such as HO‐1 and NQO1 [7, 8]. Although this response is beneficial for protecting normal tissues from oxidative injury, cancer cells can exploit persistent Nrf2 activity to maintain redox homeostasis, support proliferation, and resist therapy‐induced stress [9, 10, 11, 12]. In lung cancer models, Nrf2 activation has been implicated in enhanced growth and resistance to anticancer drugs, whereas Nrf2 inhibition can sensitize tumor cells to cytotoxic stress [13, 14, 15, 16, 17, 18, 19]. Therefore, the Nrf2/ARE pathway represents a biologically plausible target for reducing the survival capacity of NSCLC cells.

In A549 cells, sinensetin reduced both total Nrf2 and nuclear Nrf2. The decrease in ARE‐luciferase activity, normalized to co‐transfected Renilla luciferase, provided functional evidence that Nrf2‐dependent transcription was suppressed. Consistent with this result, the downstream antioxidant proteins HO‐1 and NQO1 were reduced after sinensetin treatment. Direct ROS measurements further demonstrated increased intracellular ROS accumulation, while the ability of NAC to partially reverse ROS accumulation, loss of cell viability, and apoptosis provides functional support for a redox‐dependent component of the response. These findings more directly link impaired antioxidant defense to oxidative stress than changes in pathway markers alone.

The impact of sinensetin on Nrf2 signaling should be interpreted in a context‐specific manner. In chemical injury or inflammatory models, Nrf2 activation can protect normal tissues from oxidative damage, and sinensetin has been reported to activate Nrf2 in carbon tetrachloride‐induced oxidative injury [26]. However, in established cancer cells, particularly those with high basal antioxidant capacity, the same pathway may instead favor survival and therapeutic resistance [9, 10, 11, 12, 16, 17, 18, 19]. Our data suggest that, in A549 NSCLC cells, sinensetin suppresses Nrf2/ARE‐driven antioxidant defense and promotes apoptosis, with Nrf2 restoration partially reversing these effects. Differences in cell type, disease state, treatment concentration, stress environment, and genetic background may contribute to divergent responses. Because the present study did not perform direct genomic profiling or KEAP1 mutational analysis of A549 and H1299 cells, the greater sensitivity of A549 cells cannot be attributed solely to Nrf2 dependence or KEAP1 status.

Apoptosis is a major mechanism through which anticancer agents limit tumor cell survival. The mitochondrial pathway is governed by the balance between pro‐apoptotic and anti‐apoptotic Bcl‐2 family members. Bax promotes mitochondrial outer membrane permeabilization, whereas Bcl‐2 opposes this process; once mitochondrial integrity is disrupted, caspase activation drives execution of apoptosis [29, 30, 31, 32]. In this study, sinensetin increased Bax and cleaved caspase‐3 while decreasing Bcl‐2, consistent with activation of mitochondrial apoptotic signaling. The flow cytometry results were consistent with these protein changes, showing increased Annexin V‐positive cells after sinensetin treatment.

Nrf2‐shRNA experiments further supported the involvement of Nrf2 in the observed phenotype. Nrf2 knockdown reduced HO‐1, NQO1, and Bcl‐2 expression and increased Bax and cleaved caspase‐3 levels, accompanied by reduced cell viability and increased apoptosis. Keap1 expression also increased following Nrf2 knockdown. Because Keap1 is not generally considered a direct transcriptional target of Nrf2, this change is more appropriately interpreted as an indirect or compensatory response, potentially involving feedback regulation or altered protein turnover. Complementary Nrf2 overexpression experiments further supported the functional involvement of Nrf2, as restoration of Nrf2 partially reversed the molecular and functional effects of sinensetin. The incomplete rescue suggests that additional pathways may also contribute to the overall response. The upstream mechanism by which sinensetin suppresses Nrf2, including possible effects on transcription, phosphorylation, or ubiquitin‐proteasome degradation, remains to be clarified.

Several limitations should be acknowledged. First, robust suppression of the Nrf2/ARE pathway was observed mainly at relatively high concentrations of sinensetin, which warrants cautious pharmacological and translational interpretation. Second, the present study was conducted entirely in vitro, and in vivo studies will be required to determine whether the observed antitumor effects and Nrf2/ARE modulation can be reproduced under physiologically relevant exposure conditions. Finally, the upstream molecular mechanism by which sinensetin reduces Nrf2 protein remains unresolved and requires further investigation.

## 5 Conclusion

Taken together, sinensetin reduced NSCLC cell viability and enhanced apoptosis, with A549 cells showing the strongest response. The effect was associated with suppression of Nrf2/ARE‐mediated antioxidant defense, increased intracellular ROS, and activation of mitochondrial apoptotic signaling. NAC partially attenuated ROS accumulation and apoptosis, whereas Nrf2 overexpression partially restored antioxidant signaling and cell survival. These findings support a mechanistic role for Nrf2/ARE suppression and oxidative stress in the pro‐apoptotic action of sinensetin in vitro. Further studies are needed to clarify the upstream regulation of Nrf2 and to determine whether these effects can be achieved at pharmacologically relevant exposures in vivo.

## Author Contributions

Junyan Li and Ruoyun He contributed equally to this work. Junyan Li and Ruoyun He performed most experiments and analyzed data. Jiake He, Jian Ye, and Yu Xie contributed to experimental work, data analysis, and manuscript preparation. Binfan Yuan conceived and supervised the study, interpreted data, and revised the manuscript. All authors read and approved the final manuscript.

## Ethics Statement

Ethical approval was not required for this study because only commercially available human cell lines were used and no human participants, human tissues, or animal experiments were involved.

## Conflicts of Interest

The authors declare no conflicts of interest.

## Data Availability

The datasets used and/or analyzed during the current study are available from the corresponding author on reasonable request. Raw data and analyses will be retained by the authors in accordance with Wiley data‐retention expectations.
